# Body balance on Horus® computerized posturography and body measurements in healthy children

**DOI:** 10.1590/2317-1782/e20240026en

**Published:** 2025-03-24

**Authors:** Ândrea de Melo Boaz, Rudimar dos Santos Riesgo, Pricila Sleifer

**Affiliations:** 1 Programa de Pós-Graduação em Saúde da Criança e do Adolescente, Faculdade de Medicina, Universidade Federal do Rio Grande do Sul – UFRGS - Porto Alegre (RS), Brasil.; 2 Hospital de Clínicas – HCPA, Porto Alegre (RS), Brasil.; 3 Departamento de Saúde e Comunicação Humana, Universidade Federal do Rio Grande do Sul – UFRGS - Porto Alegre (RS), Brasil

**Keywords:** Postural Balance, Child, Vestibular System, Diagnostic Equipment, Body Mass Index

## Abstract

**Purpose:**

To verify possible associations between body balance and growth curves, weight, height and body mass index in healthy children.

**Methods:**

Quantitative cross-sectional study. Two hundred and sixteen children aged between 4 and 6 years and 11 months participated. An interview was carried out with the parents/guardians and the children underwent visual screening, auditory assessment (pure tone audiometry screening method, otoacoustic emissions and immittance testing), verification of weight and height measurements and Horus® computerized posturography. Anthro and AnthroPlus software were used to classify the growth curves by age, sex, height, weight and body mass index. Responses were analyzed using the non-parametric Kruskal-Walli and post hoc Dunn-Bonferroni statistical tests for pairwise comparisons between ages, with p<0.05.

**Results:**

The measurement of weight by length showed a correlation with different examination conditions at 4 years-old, mainly for the average speed data. The same data, under different examination conditions, showed a correlation with the body mass index at 4 and 5 years of age, between children classified with weight outside the expected standard and those underweight. Children considered underweight had greater instability in body balance. Height and weight showed correlation for different examination conditions in the three age groups.

**Conclusion:**

There was an association between responses in posturography and individual body measurements (weight, height and body mass index. Therefore, it is suggested that these measurements be analyzed and considered in the evaluation with computerized posturography in children aged 4 to 6 years.

## INTRODUCTION

Posturography is an evaluation tool that allows a global analysis of the balance, as it integrates access conditions to visual, somatosensory and vestibular information, aiming to measure body oscillations^(1)^, objectively and precisely quantifying the relevant parameters in time and frequency domains in interpatient comparison^(1)^. It consists of a force platform, which checks the oscillations made by the subject positioned on it, in relation to the center of gravity and the center of pressure (CP)^(2)^.

In adults, it is known that the responses obtained in posturography are related to the subject's anthropometric characteristics, such as height and weight measurements, allowing interference in responses^(3)^. This demonstrates that differences in the subject's body composition and other variables can influence the results of the exam^(4)^. For clinical investigations, it is necessary to standardize and standardize assessment procedures, and care must be taken when quantifying body balance using anthropometric measurements^(3)^.

In children, there is a correlation between age and measurements such as height and weight in the child, as well as a positive correlation between age and the magnitude of body sway^(5)^. In the childhood, anthropometric measurements can negatively interfere with body balance control, although their correlation is not clearly defined^(6)^.

In the evaluation with computerized posturography of the dynamic type, there were associations between excess body weight and values lower than expected ​​in different conditions of the exam, indicating interference of anthropometric measurements in the postural balance response in children aged 6 to 7 years^(7)^. In the evaluation of overweight and obese children, between 6 and 9 years old, using dynamic posturography and force platform, a deficit in postural balance was found, which researchers believe was enhanced by the variables of sex, age, level of physical activity and anthropometric characteristics^(8)^.

It is suggested that in studies to standardize computerized posturography, analyzes of the findings using anthropometric measurements should be carried out^(9)^. Body measurements such as height and weight can influence, in addition to the age factor, the development of balance in children, as structural changes occur in the body continuously during growth, causing the sensory inputs to the vestibular system to be updated/changed throughout childhood^(10)^.

It was found in the scientific literature that there is influence of body weight measurements and height on postural balance in children, however, no studies were found seeking the correlation between body measurements in children and the findings in the Horus® computerized posturography, which justifies the relevance of the present work. Thus, the objective is to verify the existence of an association between body balance (through the findings of Horus® computerized posturography) and body measurements through growth curves for age, sex, height, weight and body mass index (BMI) in healthy children, aged 4 to 6 years and 11 months.

## METHODS

The study described is cross-sectional, using a quantitative approach. It was approved by the Ethics Committee of the Federal University of Rio Grande do Sul (UFRGS), with opinion number 39835. Furthermore, the study followed the ethical standards and guidelines established by Resolution 466/12 of the National Health Council, which regulates research involving human beings.

During the process of recruitment and participation in the study, strict measures were taken to ensure informed consent from participants. The Informed Consent Form was provided to those responsible for the children, explaining in detail the objectives, procedures, risks and benefits of the research. This term was signed by those responsible to authorize the child's participation in the study.

Additionally, acceptance of the Child Assent Form was requested, allowing those children who were already capable of understanding the process to consent individually, by signing, when applicable. In addition, consent for image use was obtained, ensuring that any visual recordings of children during the study were used in accordance with established guidelines.

Regarding the inclusion criteria, children should be between 4 and 6 years and 11 months old, present typical development according to the stages of child development and not present otoneurological and/or auditory complaints. The children who were unable to participate, that is, those who met the exclusion criteria, were those with a body weight of less than 15 kg; diagnosis of syndromes, hearing loss or otological changes; presence of craniofacial abnormalities; complaints of headaches, vertigo, motion sickness; history of frequent falls or presence of dizziness; visual difficulty and/or motor impairment which could compromise the assessment; neurological, cognitive alterations and/or evident psychological disorders; use of medications that act on the vestibular system or central nervous system; difficulty in understanding or, for any reason, to carry out the procedures and complete all assessment stages.

The initial contact followed by taking an anamnesis with the child's parents/guardians, in a self-report format, aiming to understand the child's development to date, as well as the presence of previous diagnoses and/or disorders, verification of possible auditory complaints and /or related to body balance that could be a reason for exclusion from participating in the research. Regarding child development, the domains of language and speech, cognitive, social, emotional, gross and fine motor were explored in detail.

Anthropometric measurements of height and weight were measured using a measuring tape fixed to the wall of the examination room and using a digital scale available on site, respectively, with body weight later confirmed using the measuring scale present on the Horus® platform. Aiming to analyze these measures, data were added individually, for each participant, in the Anthro software^(11)^ for children under 5 years of age and in the AnthroPlus software^(12)^ for those over 5 years of age, both programs available free of charge, online, by the World Health Organization (WHO). For data registration and analysis, it was also necessary to manually add data on date of birth and measurement date.

After including these data, the software generates graphs containing response curves for the variables weight for height, weight for age, height for age and body mass index (BMI) for age, in Anthro^(11)^ and, for the variables weight for age, height by age and BMI by age, in AnthroPlus^(12)^. Therefore, the answers presented in Standard Deviation (SD) can be interpreted through the different colors present in the lines of the graphs: green for within normal range (value within the population mean), adequate value; yellow for SD -1 and SD +1 being considered a warning sign; red for SD -2 and SD +2 referring to a possible nutritional problem; and black for SD -3 and SD +3 asking for data confirmation^(13)^ and purple for biological implausibility (BI). BI refers to when the child is much taller and/or weight for age, thus suggesting an implausible value for the registered subject, however, when present, the child's measurements were reassessed to confirm the data and if correct, the classification was maintained.

For body mass index (BMI), in children up to 5 years old, the data is interpreted as follows: SD +1 are described as "at risk of overweight", above SD +2 as overweight, and above SD+3 as obese^(14)^; and for children over 5 years: SD+1 refers to overweight, SD+2 refers to obesity, SD +3 to severe obesity, SD -1 refers to underweight, SD -2 refers to if thinness, SD -3 for extreme thinness and SD for a value within the normal range^(13)^.

According to the guidelines of the AnthroPlus Software User's Manual^(14)^, the creation of two programs was necessary due to the value of the cutoff point for the classification of overweight and obesity, after analyzing the populations, being different in the age groups in the two age groups, age under 5 years and range from 5 to 19 years. The classification of nutritional status is based on these cutoff points, established by the WHO for weight for age, height for age indices and BMI for age^(14)^.

Subsequently, the battery of tests that formed part of the evaluations began with the evaluation of the peripheral auditory system, with visual inspection of the external auditory canal using a Welch Allyn otoscope, tonal audiometry screening method which consists of sweeping^(15)^ at an intensity of 20 dB in the frequencies of 500, 1000, 2000 and 4000 Hz in both ears, individually evaluated. Audiometry was performed using Callisto equipment and TDH-39 model headphones, with the child sitting comfortably in a chair positioned inside an acoustic booth; Transient otoacoustic emissions (TEOAE) of the screening type, using AccuScreen equipment and the use of insertion-type headphones; Tympanometry with a 226Hz probe and investigation of contralateral acoustic reflexes at frequencies of 500, 1000, 2000 and 4000 Hz. These were carried out with Zodiac equipment and the use of insertion and probe type headphones. Those children who presented responses present in TEOAE and in all frequencies tested in pure tone audiometry, type A tympanometric curve and acoustic reflexes present in both ears were considered eligible. All equipment used in this study was calibrated according to ISO 8253-1 standards.

The assessment of the visual system was carried out through visual acuity screening using the Snellen “E” directional optotype, following the guidelines provided by the Ministry of Health^(16)^. This test is performed by evaluating each eye individually. To do this, the child wore an eye patch with a children's illustration and in a playful way the term: "like a pirate" was used. They were asked to cover the left eye that was not being evaluated at the time, and then cover the right eye, being observed and checked by the examiner whether it was really covered and without pressure on the eyes. For the assessment, the child was asked to speak or point in which direction they were seeing the "legs of the letter E", previously explained and demonstrated. The evaluation began by showing the figures from top to bottom on the sheet, positioned on the wall of the room at a distance according to the Ministry of Health guidelines^(16)^, that is, the largest letters were presented first to the smallest. Responses were considered to be within the range of vision, that is, correct responses corresponding to line 10.

Later, body balance was analyzed using computerized posturography Horus®, from the company Contronic. There was a need to adapt the platform and software by the manufacturer, especially adapted for this research, due to the need to adapt the minimum acceptable weight to carry out the tests. To the evaluation, the “Power Platform” was connected via USB to an Asus X450CA notebook, which contains the Horus® software that displays and records all data provided. The images, necessary for carrying out the evaluation, were presented on a 40-inch Sony television set at a distance of 1 meter from the platform and the child's positioning point, located so that the screen was at eye level. As for physical height measurements, the platform is 5cm high and the foam cushion is 5cm high with density D33, which was added to carry out test conditions 3 to 7.

Regarding the guidelines and initial positioning: the child was barefoot, climbed onto the platform, remained standing, with feet apart and remained motionless, as still as possible. It is worth noting that the feet should be aligned on the horizontal line under the platform, symmetrically distanced from the anteroposterior line, with the hallux toe pointing between 0 and 15 degrees. Furthermore, he/she was instructed to remain still, with his/her eyes open, looking at the television where images would appear, and, finally, when warned, he/she should close her eyes. The complete posturography assessment consisted of eight examination conditions, lasting 30 seconds each, with the child being specifically guided in each condition. The description of the evaluation conditions can be seen in Chart 1.

**Chart 1:** Evaluation conditions of the Horus® Computerized Posturography exam

CONDITION	SYSTEM EVALUATED	GUIDANCE FOR EXECUTION
Stability Limit (SL) - Fixed platform, without pad	Forms the stability area of the body	Climb onto the platform and lean body forward, returning to the center, lean back, returning to the center, lean body to the right, returning to the center, lean body to the left, returning to the center and then repeat. It should be performed without rushing, without moving hips or shoulders and without removing feet from the platform, moving only the ankles. Longest stage, due to the need to learn the movement and repetitions.
Sensory condition 1 (C1) - Fixed platform, without pad	Somatosensory, visual and vestibular	Stand with eyes open, looking at a fixed yellow dot of a 10% size against a black background, avoiding moving.
Sensory condition 2 (C2) - Fixed platform, without pad	Somatosensory and vestibular	Stand with eyes closed without moving.
Sensory condition 3 (C3) - Platform with pad	Somatosensory and visual	Same orientation of sensory condition 1
Sensory condition 4 (C4) - Platform with pad	Vestibular	Same orientation of sensory condition 2
Sensory condition 5 (C5) - Platform with pad and visual conflict	Visual preference, conflict between visual and vestibular systems. Somatosensory imprecision.	Remain standing, without moving, watching television watching a video containing an image of black and white bars in horizontal optokinetic effect to the right, at a speed of 10%.
Sensory condition 6 (C6) - Platform with pad and visual conflict	Visual preference, conflict between visual and vestibular systems. Somatosensory imprecision.	Remain standing, without moving, looking at the television showing a video containing an image of bars in black and white in horizontal optokinetic effect to the left, at a speed of 10%.
Sensory condition 7 (C7) - Platform with pad and visual conflict	Visual preference, conflict between visual and vestibular systems. Somatosensory imprecision.	Remain standing, without moving, watching a video on television of a tunnel made up of thin bars moving forward at a speed of 4%, without rotation.

To facilitate the assessment with this audience, it was necessary to use some playful strategies, depending on the child's individual needs at the time. These strategies included: inviting the child's companion to imitate the activity, positioning themselves next to the child; use a “Mr. Potato” doll standing like a statue on the ground next to the platform; count the time out loud, in ascending or descending order; suggest that, in test conditions containing image presentation, an animal could appear at the end of the time, making it necessary to remain still so as not to scare the animal and make it go away (for example: a fixed yellow dot on the television screen in exam condition 1 could transform into a chick at the end of 30 seconds and/or a zebra could appear at the end of the time in the evaluation with the presentation of an optokinetic stimulus). These strategies helped maintain children's attention and cooperation during the assessment, especially younger children.

For each child, a second person was always present, positioned close to them during the examination, aiming to provide security and confidence. Furthermore, the study had two evaluators to improve the reliability of the findings, checking the responses and positioning/behavior of the child during the posturography evaluation.

At the end, with all posturography examination conditions evaluated, the values ​​of postural oscillations were recorded by the stabilogram in the direction of movement to the left and right (medio-lateral) and forward and backward (anteroposterior). Data were also recorded by the statokinesigram, which documents the displacement of the body in the CP to the mediolateral axis and in relation to the displacement of the CP in the anteroposterior axis, with a 95% confidence ellipse.

In this work, the following data obtained from Horus® posturography were listed for study: a) Stability Limit (SL), which refers to the distance at which the child was able to move in relation to the center of gravity, without having to change the support base; b) SL area, is the region in which the body moved without falling/imbalance, in mm^2^; c) Confidence ellipse (CE), is quantitatively the amount of displacement/difficulty the child had in remaining without oscillation in relation to the CP, in mm^2^; d) Speed ​​that represents the average speed in relation to CP. A lower average speed (in mm/s) is interpreted as indicating better body balance; e) Residual Functional Balance (RFB), is the region still available for oscillation, safely, due to the relationship between the SL area and the area of the EC, expressed as a percentage. The higher the RFB value, the better the body balance.

If any change was identified in any of the assessment stages, regardless of the examination carried out, the parents/guardians were advised and the child was referred for additional specialized care.

Data were analyzed using the Statistical Package for Social Sciences (SPSS) software for Windows version 22.0. Categorical data were presented in relative frequency, while quantitative data were described by mean and standard deviation. To compare the different age groups, the Kruskal-Wallis nonparametric statistical tests and the Dunn-Bonferroni post hoc test were used for pairwise comparisons. Differences were considered statistically significant when p < 0.05.

## RESULTS

Initially, 231 children participated in the research, however, considering the eligibility criteria, the final sample was 216 children. Of these, 77 were between 4 years and 4 years and 11 months, 69 were between 5 years and 5 years and 11 months, and 70 were in the age group of 6 years and 6 years and 11 months.

**Figure 1:**
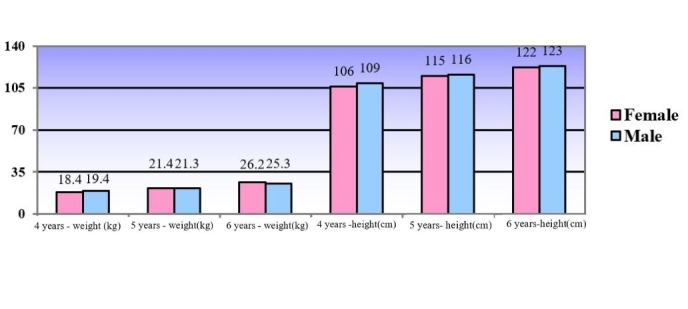
Average weight, in kilograms, and height, in centimeters, of the children participating in the research (n=216)

Regarding the anthropometric values of height, in centimeters, and weight, in kilograms, for each age group, are shown in Figure 1.

**Caption:** Kg = kilogram; cm = centimeters

There was a statistically significant finding in the relationship between height measurement at 4 years of age (p=0.029). In the analysis of the weight by length measurement with the posturography responses, there was no statistical difference for the stability limit. However, statistical significance was observed in the first three conditions of the examination in 4-year-old children, as described in Table 1.

**Table 1:** Data from the measurement of weight by length and the responses in the different conditions of the posturography exam in 4-year-old children (n=77)

	N	Average± S.D.	Minimum	Percentile 05	Percentile 25	Median	Percentile 75	Percentile 95	Maximum	p-value*	Pairwise **
CONDITION 1 - FIXED PLATFORM AND OPEN EYES											
AVERAGE LATERAL VELOCITY mm/s											
SD (A)	48	10.4± 4.7	4.0	4.5	6.5	8.6	14.5	19.3	21.7	0.028	D≠E 0.028
SD+1(B)	8	-4.1± 5.9	-11.0	-11.0	-10.1	-3.9	1.2	3.4	3.4		
SD+2(C)	6	8.4± 2.5	5.6	5.6	7.1	7.5	10.2	12.5	12.5		
BI (D)	11	7.1± 2.4	3.5	3.5	5.2	7.2	9.2	11.3	11.3		
SD-1 (E)	4	15.1± 4.1	10.5	10.5	12.0	14.9	18.3	20.2	20.2		
MAXIMUM LATERAL AVERAGE DISPLACEMENT TO THE LEFT mm											
SD (A)	48	-21.3± 13.3	-59.9	-48.5	-29.1	-21.6	-12.0	-2.7	2.2	0.024	C≠E 0.047
SD+1(B)	8	-19.5± 12.4	-37.7	-37.7	-30.1	-18.5	-9.4	-2.6	-2.6		
SD+2(C)	6	147.8± 392.3	-26.1	-26.1	-18.2	-8.8	0.2	948.3	948.3		
BI (D)	11	-12.9± 10.4	-26.8	-26.8	-24.0	-12.6	-3.0	0.6	0.6		
SD-1 (E)	4	-33.7± 9.9	-47.9	-47.9	-39.7	-30.9	-27.7	-25.1	-25.1		
CONDITION 2 - FIXED PLATFORM AND EYES CLOSED											
CONFIDENCE ELLIPSE mm^2^											
SD (A)	48	1016.9± 634.1	100.5	244.7	574.3	894.9	1244.0	1957.5	3301.8	0.025	D≠E 0.041
SD+1(B)	8	995.9± 845.4	223.0	223.0	493.7	725.2	1260.9	2784.4	2784.4		
SD+2(C)	6	693.9± 624.0	168.0	168.0	412.2	513.8	628.7	1926.7	1926.7		
BI (D)	11	598.0± 385.3	224.1	224.1	299.7	449.3	854.7	1325.7	1325.7		
SD-1 (E)	4	1521.4± 287.1	1181.7	1181.7	1283.7	1565.3	1759.1	1773.3	1773.3		
CONFIDENCE ELLIPSE BY STABILITY LIMIT %											
SD (A)	48	12.5± 6.4	1.3	4.0	7.0	11.2	17.9	24.3	25.2	0.027	-
SD+1(B)	8	9.3± 3.9	5.2	5.2	6.5	8.5	12.0	15.7	15.7		
SD+2(C)	6	8.4± 5.7	2.3	2.3	2.8	8.3	11.3	17.6	17.6		
BI (D)	11	8.5± 5.4	2.9	2.9	3.5	7.7	9.6	21.6	21.6		
SD-1 (E)	4	19.2± 6.1	11.5	11.5	14.7	19.7	23.7	25.9	25.9		
AVERAGE LATERAL VELOCITY mm/s											
SD (A)	48	12.9± 4.9	3.5	5.8	8.8	12.4	16.8	19.9	28.3	0.002	C≠E 0.017 D≠E 0.003
SD+1(B)	8	12.7± 5.5	6.8	6.8	10.0	11.5	13.2	25.2	25.2		
SD+2(C)	6	9.2± 2.8	5.7	5.7	6.7	9.0	11.4	13.3	13.3		
BI (D)	11	8.9± 3.0	5.6	5.6	6.6	8.1	9.9	15.8	15.8		
SD-1 (E)	4	22.5± 6.2	15.9	15.9	17.4	22.1	27.5	29.7	29.7		
AVERAGE ANTEROPOSTERIOR VELOCITY mm/s											
SD (A)	48	21.6± 6.4	9.8	11.5	16.4	22.1	25.3	32.5	35.5	0.028	D≠E 0.026
SD+1(B)	8	19.1± 4.1	14.4	14.4	16.0	18.4	22.0	25.7	25.7		
SD+2(C)	6	19.1± 6.1	12.5	12.5	14.2	18.1	22.6	29.4	29.4		
BI (D)	11	18.0± 5.1	11.2	11.2	15.2	17.1	21.0	29.7	29.7		
SD-1 (E)	4	29.7± 4.4	24.3	24.3	26.3	30.2	33.2	34.2	34.2		
RESIDUAL FUNCTIONAL BALANCE %											
SD (A)	48	87.5± 6.4	74.8	75.7	82.1	88.9	93.0	96.0	98.7	0.027	-
SD+1(B)	8	90.7± 3.9	84.3	84.3	88.1	91.6	93.5	94.8	94.8		
SD+2(C)	6	91.6± 5.7	82.4	82.4	88.7	91.8	97.2	97.7	97.7		
BI (D)	11	91.5± 5.4	78.4	78.4	90.4	92.3	96.5	97.1	97.1		
SD-1 (E)	4	80.8± 6.1	74.1	74.1	76.3	80.3	85.3	88.5	88.5		
TOTAL AVERAGE VELOCITY mm/s											
SD (A)	48	26.4± 8.5	10.2	12.9	20.7	25.3	31.8	41.8	45.3	0.011	D≠E 0.009
SD+1(B)	8	24.4± 7.2	15.9	15.9	20.2	23.0	26.9	39.3	39.3		
SD+2(C)	6	21.7± 7.0	14.4	14.4	15.2	20.8	25.9	33.1	33.1		
BI (D)	11	21.1± 6.3	13.1	13.1	17.6	19.2	23.1	35.9	35.9		
SD-1 (E)	4	39.8± 6.8	31.0	31.0	35.5	40.3	44.1	47.6	47.6		
CONDITION 3 - PLATFORM WITH CUSHION AND OPEN EYES											
LATERAL AVERAGE VELOCITY mm/s											
SD (A)	48	14.3± 4.8	5.6	7.0	11.1	13.5	18.3	22.7	25.4	0.017	C≠E 0.005
SD+1(B)	8	14.8± 5.6	8.5	8.5	10.2	13.2	20.1	23.0	23.0		
SD+2(C)	6	9.9± 3.3	5.3	5.3	6.5	11.0	11.2	14.1	14.1		
BI (D)	11	14.0± 3.9	7.8	7.8	10.5	13.0	17.7	20.4	20.4		
SD-1 (E)	4	21.4± 1.7	19.0	19.0	20.4	21.8	22.5	23.1	23.1		
TOTAL AVERAGE VELOCITY mm/s											
SD (A)	48	25.3± 7.6	9.5	12.5	20.6	23.3	30.0	39.7	42.4	0.032	C≠E 0.012
SD+1(B)	8	25.9± 9.0	14.6	14.6	19.1	23.9	33.4	39.6	39.6		
SD+2(C)	6	18.9± 5.5	12.3	12.3	13.3	19.1	23.6	26.0	26.0		
BI (D)	11	25.6± 4.9	19.9	19.9	22.2	23.8	30.3	34.4	34.4		
SD-1 (E)	4	36.5± 4.6	30.7	30.7	33.0	37.0	40.0	41.2	41.2		
SENSORY ANALYSIS VISUAL PERCEPTION											
SD (A)	48	96.5± 6.1	68.6	88.5	93.3	96.4	100.9	104.2	105.8	0.04	C≠D 0.033
SD+1(B)	8	97.6± 3.5	94.2	94.2	94.8	96.0	101.0	102.7	102.7		
SD+2(C)	6	100.7± 4.9	94.4	94.4	97.5	99.9	105.3	107.2	107.2		
BI (D)	11	93.2± 3.8	85.5	85.5	90.8	94.6	96.1	98.0	98.0		
SD-1 (E)	4	97.4± 2.5	93.7	93.7	96.0	98.4	98.8	98.9	98.9		
SENSORY ANALYSIS VESTIBULAR PERCEPTION											
SD (A)	48	89.7± 7.8	71.6	73.7	85.7	89.0	95.4	102.2	106.2	0.027	C≠D 0.029
SD+1(B)	8	90.8± 6.5	82.2	82.2	84.9	92.0	94.9	101.1	101.1		
SD+2(C)	6	93.9± 5.3	85.9	85.9	91.1	93.7	99.1	100.2	100.2		
BI (D)	11	83.5± 4.5	76.7	76.7	79.8	83.2	88.3	91.0	91.0		
SD-1 (E)	4	90.4± 11.0	78.5	78.5	81.2	90.8	99.7	101.5	101.5		

P*: P-value referring to the Kruskal-Wallis nonparametric statistical test and the Dunn-Bonferroni post hoc test were used for pairwise age comparisons

* , ** refer to statistically significant values

**Caption:** N: volunteers; S.D.: standard deviation; SD for value within normal range; SD +1 refers to overweight; SD +2 refers to obesity; BI biological implausibility. refers to when much higher than expected for age; SD -1 refers to underweight (as categorized by WHO Anthro^(11)^ software)

In the analysis of the height variable, there was a statistical difference for C1 in the medio-lateral CP (p=0.039, with SD +1 vs. normal p= 0.035), for C3 in the anteroposterior CP (p=0.047, being SD +1 vs. SD +2 p= 0.036 and +2 vs. normal p= 0.032), for C4 in the medio-lateral mean velocity (p=0.014, being SD + 1 vs. normal p= 0.029), C5 in compound equilibrium (p=0.008, with SD +1 vs. SD -1 p= 0.041, SD +2 vs. SD -1 p= 0.012, SD -1 vs. BI p = 0.03) and for average medio-lateral speed (p=0.011, no pair-to-pair relationship) and total (p= 0.022, no pair-to-pair relationship), C6 for average medio-lateral speed (p=0.01 , without pairwise relationship), anteroposterior (p=0.014, SD +1 vs. normal p= 0.013) and total (p= 0.006, SD +1 vs. normal p= 0.017), and C7 for composite balance measurements (p=0.007, with SD -1 vs. normal p= 0.037, SD -1 vs. SD +1 p= 0.009, SD -1 vs. BI p= 0.014), anteroposterior center of pressure (p= 0.028, with SD +2 vs. normal p= 0.035, SD +1 vs. SD +2 p= 0.013), mediolateral velocity (p=0.49, no pairwise relation).

Below are the responses with significant results, by age, according to the variables of weight, height and BMI:

Regarding weight: At 4 years of age, a statistical difference was observed for C4 in average anteroposterior velocity (p=0.014, without pairwise relationship) and for C7 in maximum mediolateral displacement to the left (p=0.05, without pairwise relationship) and to the right (p=0.023, with SD +2 vs. BI p= 0.033); at 5 years old, there was a difference in SL (p=0.023, with SD -1 vs. normal p= 0.043 and SD -1 vs. BI p= 0.019) and for C5 in the average medio-lateral velocity (p=0.023, no relation pairwise).

Regarding height: At 4 years of age, there was a statistically significant result for the vestibular sensory analysis (p=0.043 without pairwise relationship), SL in the maximum medio-lateral displacement to the right (p= 0.006, being SD-1 vs. normal p= 0.018); at 5 years of age, the results were significant for the SL measurement (p=0.004, no pairwise relationship), for C3 in the average mediolateral velocity (p= 0.024, with SD +1 and SD -1 p=0.040) and in the maximum lateral displacement to the left (p= 0.048, without pairwise relationship), for C4 in the average mediolateral velocity (p= 0.025, without pairwise relationship), for C5 in compound balance (p= 0.028, without pair-to-pair relationship), and for C6 in the measurements of average medio-lateral (p= 0.035, no pair-to-pair relationship), anteroposterior (p= 0.025, no pair-to-pair relationship) and total (p= 0.015, being SD +1 vs. SD -1 p= 0.045); at 6 years old, for the SL measurement (p=0.024, without pairwise relationship), for C4 in the composite balance measurements (p= 0.026 without pairwise relationship) and average mediolateral speed (p= 0.017, being SD +2 vs. normal p= 0.018) and total (p= 0.013, with SD +2 vs. normal p= 0.007), for C5 in the medial-lateral velocity (p= 0.04 without pairwise relationship), and for C6 in average medio-lateral velocity (p= 0.019, being SD +1 vs. normal p= 0.024) and total (p= 0.031, being SD +1 vs. normal p= 0.022), and maximum medio-lateral displacement to the right (p= 0.011 without pairwise relationship).

Regarding BMI: There was no significant difference in any of the analyzes for 6-year-old children. However, for 5-year-old children, BMI showed a relationship with the average total speed for the SL (p=0.05, with SD -1 vs. BI p=0.048). For the 4-year age group, more significant results were observed, as can be seen in Table 2.

**Table 2:** Body mass index values and responses in different assessment conditions in posturography in 4-year-old children (n=77)

	N	Average ± S.D.	Minimum	Percentile 05	Percentile 25	Median	Percentile 75	Percentile 95	Maximum	p-value*	Pairwise**
CONDITION 1 - FIXED PLATFORM AND OPEN EYES											
AVERAGE LATERAL VELOCITY mm/s											
SD (A)	51	10.4± 4.6	4.0	4.2	6.3	9.2	14.4	19.3	21.7	0.033	D≠E 0.032
SD+1(B)	9	10.1± 4.1	4.7	4.7	7.6	8.4	12.4	16.5	16.5		
SD+2(C)	5	8.0± 2.6	5.6	5.6	7.1	7.2	7.4	12.5	12.5		
BI (D)	9	7.2± 2.3	3.5	3.5	6.2	7.2	7.5	11.3	11.3		
SD-1 (E)	3	16.7± 3.4	13.5	13.5	13.5	16.3	20.2	20.2	20.2		
MAXIMUM AVERAGE DISPLACEMENT TO THE LEFT											
SD (A)	51	-21.7± 13.0	-59.9	-48.5	-29.4	-22.0	-12.6	-2.7	2.2	0.004	A≠C 0.023 C≠E 0.011
SD+1(B)	9	-18.5± 11.8	-37.7	-37.7	-25.9	-17.6	-10.9	-2.6	-2.6		
SD+2(C)	5	185.6± 426.4	-16.7	-16.7	-3.0	-0.8	0.2	948.3	948.3		
BI (D)	9	-13.1± 10.1	-24.0	-24.0	-21.7	-18.2	-3.5	0.6	0.6		
SD-1 (E)	3	-34.8± 11.8	-47.9	-47.9	-47.9	-31.5	-25.1	-25.1	-25.1		
CONDITION 2 - FIXED PLATFORM AND EYES CLOSED											
CONFIDENCE ELLIPSE mm^2^											
SD (A)	51	1010.4± 622.9	100.5	244.7	567.0	885.8	1248.0	1957.5	3301.8	0.016	A≠D 0.046 D≠E 0.027
SD+1(B)	9	1013.8± 796.0	223.0	223.0	603.1	731.5	1212.5	2784.4	2784.4		
SD+2(C)	5	796.9± 637.1	401.7	401.7	480.2	547.4	628.7	1926.7	1926.7		
BI (D)	9	474.0± 359.7	168.0	168.0	275.9	312.5	498.5	1325.7	1325.7		
SD-1 (E)	3	1566.6± 333.7	1181.7	1181.7	1181.7	1744.9	1773.3	1773.3	1773.3		
CONFIDENCE ELLIPSE BY STABILITY LIMIT %											
SD (A)	51	12.3± 6.1	1.3	4.0	7.0	10.6	17.6	24.0	25.2	0.026	D≠E 0.046
SD+1(B)	9	10.9± 6.2	5.2	5.2	6.6	8.2	14.8	24.3	24.3		
SD+2(C)	5	9.3± 5.6	2.8	2.8	6.9	7.7	11.3	17.6	17.6		
BI (D)	9	7.2± 5.9	2.3	2.3	3.4	6.8	8.5	21.6	21.6		
SD-1 (E)	3	19.6± 7.4	11.5	11.5	11.5	21.5	25.9	25.9	25.9		
ANTEROPOSTERIOR PRESSURE CENTER mm/s											
SD (A)	51	42.8± 12.8	8.8	23.2	36.2	41.0	48.5	64.3	82.2	0.048	A≠D 0.049
SD+1(B)	9	37.8± 5.6	30.1	30.1	34.7	37.4	39.2	50.0	50.0		
SD+2(C)	5	37.8± 4.7	32.3	32.3	35.4	36.7	40.1	44.7	44.7		
BI (D)	9	31.7± 12.2	17.4	17.4	21.4	32.5	34.9	57.2	57.2		
SD-1 (E)	3	46.6± 14.6	35.9	35.9	35.9	40.7	63.2	63.2	63.2		
AVERAGE LATERAL VELOCITY mm/s											
SD (A)	51	12.8± 4.9	3.5	5.8	8.7	12.1	17.0	19.9	28.3	0.003	A≠D 0.029 D≠E 0.004
SD+1(B)	9	13.2± 5.2	6.8	6.8	10.6	12.4	13.8	25.2	25.2		
SD+2(C)	5	9.9± 2.5	6.7	6.7	8.8	9.4	11.4	13.3	13.3		
BI (D)	9	8.2± 3.2	5.6	5.6	6.2	7.4	8.6	15.8	15.8		
SD-1 (E)	3	23.6± 7.0	15.9	15.9	15.9	25.3	29.7	29.7	29.7		
RESIDUAL FUNCTIONAL BALANCE %											
SD (A)	51	87.7± 6.1	74.8	76.0	82.4	89.4	93.0	96.0	98.7	0.026	D≠E 0.046
SD+1(B)	9	89.1± 6.2	75.7	75.7	85.2	91.8	93.4	94.8	94.8		
SD+2(C)	5	90.7± 5.6	82.4	82.4	88.7	92.3	93.1	97.2	97.2		
BI (D)	9	92.8± 5.9	78.4	78.4	91.5	93.2	96.6	97.7	97.7		
SD-1 (E)	3	80.4± 7.4	74.1	74.1	74.1	78.5	88.5	88.5	88.5		
TOTAL AVERAGE VELOCITY mm/s											
SD (A)	51	26.5± 8.5	10.2	12.9	21.1	25.2	31.7	41.8	45.3	0.02	D≠E 0.021
SD+1(B)	9	25.3± 7.2	15.9	15.9	22.3	23.7	29.3	39.3	39.3		
SD+2(C)	5	22.2± 7.5	14.4	14.4	17.0	20.5	25.9	33.1	33.1		
BI (D)	9	19.8± 6.5	13.1	13.1	17.6	18.5	20.1	35.9	35.9		
SD-1 (E)	3	39.5± 8.3	31.0	31.0	31.0	39.9	47.6	47.6	47.6		

P*: P-value referring to the Kruskal-Wallis nonparametric statistical test and the Dunn-Bonferroni post hoc test were used for pairwise age comparisons

* , ** refer to statistically significant values

**Caption:** N: volunteers; S.D: standard deviation; SD for value within normal range, SD +1 refers to overweight, SD +2 refers to obesity, BI refers to biological implausibility, refers to when much higher than expected for age, SD -1 refers to underweight (as categorized by WHO Anthro^(11)^ software)

As for nutritional status, using the Anthro^(11)^ software at 4 years of age, in girls, the weight for length indicator showed normality in 60,5% (n= 23), underweight twin sisters 5,3% (n= 2), overweight in 7,9% (n= 3), obesity in 10,5% (n= 4) and biological incompatibility in 15,8% (n= 6). In boys, normality was observed in 64,2% (n= 25), underweight in 5,1% (n= 2), overweight in 12,8% (n= 5), obesity in 5,1% (n= 2) and biological incompatibility in 12,8% (n=5).

In the BMI indicator, normality was observed in 68,4% (n= 26), underweight in 2,6% (n= 1), overweight in 5,3% (n= 2), obesity in 7,9% (n= 3) and biological incompatibility in 15,8% (n=6) in females. In males, it was observes normality in 64,2% (n= 25), underweight in 5,1% (n= 2), overweight in 17,9% (n= 7), obesity in 5,1% (n= 2) and biological incompatibility in 7,7% (n= 3);. It demonstrates that at 4 years of age, more than 1/3 of the children (n= 26) have a BMI outside the expected range for their age group.

The BMI, at the age of 5 years, analyzed by the AnthroPlus software^(12)^, demonstrated that in females, 42.9% (n= 15) of children classified as eutrophic (adequate, within normal range), 8.6% (n= 3) were underweight, 31.4% (n= 11) were overweight, 11.4% (n= 4) were classified as obese and 5.7% (n= 2) as biological incompatibility. In males, 58.8% (n= 20) were strophic, 3% (n= 1) were thin, 14.7% (n= 5) were overweight, 20.5% (n= 7) were obese and 3% (n= 1) biological incompatibility. In summary, 50.2% (n = 34) of children aged 5 years had a BMI outside the expected range for their age.

For the 6 year old age group, the BMI index (analyzed by the AnthroPlus software^(12)^) the results presented in females were: 35.3% (n= 12) eutrophic, 11.8% (n= 4) were overweight below expected, 29.4% (n= 10) overweight, 20.6% (n= 7) obesity and 2.9% (n= 1) 2.9% (n= 1) biological incompatibility. In males, 47.2% (n= 17) classified as eutrophic, 2.8% (n= 1) underweight, 25% (n= 9) overweight, 13.9% (n= 5) obese and 11.1% (n= 4) as biological incompatibility. Demonstrating that at 6 years of age, more than half of the children, 58.6% (n = 41), had a BMI outside the expected range for their age.

## DISCUSSION

Changes in postural control can generate delays in motor development and learning, resulting in interference with potential related to language, speech, writing and reading^(17)^. This reinforces the need for well-defined normality standards in assessment equipment, which consider the particularities of age and individual variables such as anthropometric measurements and gender. It also aims to add to clinical practice by using a safe tool for monitoring child development.

It is worth noting that maturation during development occurs respecting an order, being no different for the maturation of the sensory systems involved in body balance, which occurs first in the visual system, then in the proprioceptive system and followed by the vestibular system, reaching functional maturation at 9 years old^(18)^. Additionally, the postural adjustments made by the body are essential in compensating for disturbances received during movements that need to be coordinated in space. In this sense, the vestibulospinal reflex (VSR) plays an important role, as it allows coordinating the movement of the head and neck with the movement of the trunk and body, aiming to keep the head in an upright position and participating in the postural stabilization of the body globally even in the absence of active locomotion^(19)^. This highlights the relevance of research in the area of ​​pediatric Otoneurology, which seeks greater elucidation of the maturational development of body balance and possible associations with anthropometric measurements, as well as, through the use and study of updated equipment aimed at assessment, diagnosis and intervention in this public.

The measurement of weight by length is used to monitor and follow the growth and development of children up to 5 years old^(14)^. In the present study, this measure showed a significant association in 4-year-old children for data on average speed, as well as responses related to the visual and vestibular systems, possibly due to the statistical differences observed in the height of children in this age group. The oscillation values observed in the average speed were higher in children classified as SD-1, underweight, compared to those classified as SD+2 and/or BI, overweight. It is assumed that this is due to the slower body movement, which may be related to the increase in body mass and, consequently, to the reduction in adjustment oscillation in children considered heavier. This contributes to greater stability of the body in relation to the center of pressure.

A study^(20)^ verified the possible correlation between the responses of the center of pressure on a force platform and the variable overweight versus normal weight, in children and adolescents, aged between 7 and 12 years. The results revealed that there was no significant difference between pairs of the same age in the four sensory conditions evaluated. However, there was greater variability in responses in those who were overweight, for the speed data, more specifically for the mediolateral speed^(20)^. In the present study, in the first examination condition, a statistically significant difference was observed in the mediolateral speed between the extremes SD-1 (low weight for height) and BI (child with anthropometric values ​​much higher than expected for the age group), suggesting that weight and height influence the balance of children at 4 years of age.

Another study^(21)^ conducted with children aged 4 to 5 years showed that body weight did not have a statistically significant effect on responses to the Tandem gait and single-leg stance tests, which assess the child's ability to maintain balance on a narrow support base. However, the same variable showed a strong association with responses in dynamic and static posturography in 93 young males aged 10 to 21 years^(22)^. The researchers suggested that the changes in balance observed were too subtle to be detected by the test; furthermore, they reported as another hypothesis the reduction in muscle composition in relation to weight, and not necessarily due to changes in proprioception^(22)^. Unlike the present study, in which significant results were found in some of the examination conditions for all age groups, in the analysis of weight and body balance responses, demonstrating the influence of this variable on the control of body stability. These discrepant results can possibly be attributed to the differences in the evaluation methods compared to the first study and to the differences in the age range of the participants in the second study.

The results of this research partially corroborate another study^(23)^, which describes an association between the responses obtained on different force platforms with the weight measurement, with excess weight being significant for the anteroposterior frequency and mediolateral velocity data at 8 years of age. Researchers^(24)^ suggest that weight is a predictive factor of the responses obtained in the assessment of balance in children aged 3 to 4 years, as observed in the Functional Reach Test, which aims to identify dynamic changes in postural control, ceasing to be a predictor at age 5. The existence of a relationship between body weight and the findings in posturography is reiterated. However, the findings of the present study differ from other studies with young population, since children with underweight were those who performed worse, demonstrating less body stability compared to those who were overweight. One of the hypotheses raised is the age group studied.

Body measurements of weight and height are negatively related to postural control^(25)^. Although it is challenging to find scientific literature that explicitly exposes the impact of height and weight measurements on postural stability, researchers have observed a correlation with statistically significant dependence between the measurements and the parameters analyzed, both with eyes open and closed, in schoolchildren aged 6 to 9 years^(26)^. Height can negatively interfere with children's postural control^(6)^. After analyzing the responses from the Modified Clinical Sensory Integration Test and the results from the Balance Master posturography for the SL, an association was identified between the findings and the variables of age, sex, height and BMI in children aged 4 to 12 years^(27)^. The current findings corroborate these findings, as statistically significant differences were found across different examination conditions for the variables analyzed across all age groups.

At 6 years of age, when more than half of the children had altered BMI, no relationship was found between BMI and the conditions of the posturography exam. However, at 5 years of age, a relationship was observed in only one condition (speed) and for several variables at 4 years of age, especially for the average speed data. The relation between the average velocity measurement, which allows determining the speed of displacements of the center of pressure in posturography, was observed to be different at both 5 and 4 years of age, corroborating the results of a previous study^(6)^. These findings are consistent with a recent literature review, which concluded that the increase in oscillation of body stability is associated with a high BMI value, highlighting weight as a strong predictor of changes in body stability, according to publications in several scientific journals^(28)^.

In the present study, it could be seen that with increasing age, there was also an increase in the percentage of children with inadequate BMI. Regarding the findings on the nutritional issue, related to BMI, authors in the field of nutrition identified a tendency towards overweight in children aged 5 to 9 years, living in a small town of Rio Grande do Sul, reinforcing the observation of a nutrition disorder in the study population^(29)^. In another more populous city in the same state, a different study found that as age increased, the number of children classified as obese also increased^(30)^. There was a higher prevalence of overweight children among those aged 5 years or older compared to younger children^(30)^.

It is believed that the lack of activity, the increase in time at home, the limitations of spaces for circulation, added to the restrictions on coexistence, caused by the Coronavirus pandemic that began in 2019, may have contributed to the findings, as the collection was held from November 2020 to April 2021. This fact may have been one of the limitations of this study. It is suggested that further research be carried out on children in the same age group, especially those with vestibular pathologies and/or complaints, incorporating new findings and technological advances for an increasingly precise and comprehensive assessment.

Finally, it is reiterated that body balance is one of the essential requirements for motor learning, performance of daily activities, adequate social interaction and full development. The integration of the vestibular system with other sensory systems is important for the adequate development of complex spatial behaviors and cognitive skills by the child^(31,32)^. Changes in body balance and posture can restrict the child's adequate interaction with the environment, being able to interfere with the acquisition and development of language and learning, causing a deleterious impact on cognition, psychosocial and educational skills.

## CONCLUSION

In view of the above, it is concluded that it was possible to verify the association of individual body measurements of weight, height and BMI with the maintenance of body balance in healthy children aged 4 to 6 years and 11 months. It is recommended that such measurements be analyzed and considered in computerized posturography exams in the child population, due to their significant influence on the results obtained.
