# Supplementary material for: Body balance on Horus® computerized posturography and body measurements in healthy children
Source: Codas. 2025 Mar 24;37(2):e20240026. doi: 10.1590/2317-1782/e20240026en (PMC11938922; doi:10.1590/2317-1782/e20240026en)
Supplement: Translation

# Translation: Equilíbrio corporal na posturografia computadorizada Horus® e medidas corporais em crianças hígidas

**DOI:** 10.1590/2317-1782/e20240026pt

**Authors:** Ândrea de Melo Boaz, Rudimar dos Santos Riesgo, Pricila Sleifer

**Affiliations:** 1 Programa de Pós-Graduação em Saúde da Criança e do Adolescente, Faculdade de Medicina, Universidade Federal do Rio Grande do Sul – UFRGS - Porto Alegre (RS), Brasil.; 2 Hospital de Clínicas – HCPA, Porto Alegre (RS), Brasil.; 3 Departamento de Saúde e Comunicação Humana, Universidade Federal do Rio Grande do Sul – UFRGS - Porto Alegre (RS), Brasil.; **Correspondence address:** Ândrea de Melo Boaz Programa de Pós-graduação em Saúde da Criança e do Adolescente, Faculdade de Medicina, Universidade Federal do Rio Grande do Sul – UFRGS Rua Ramiro Barcelos, 2400, Sala 220, Porto Alegre (RS), Brasil, CEP: 900 35003 E-mail: andrea.de.melo@hotmail.com

**Keywords:** Equilíbrio Postural, Criança, Sistema Vestibular, Equipamentos para Diagnóstico, Índice de Massa Corporal

## Abstract

**Objetivo:**

Verificar possíveis associações entre o equilíbrio corporal e curvas de crescimento, peso, altura e índice de massa corporal em crianças hígidas.

**Método:**

Estudo transversal quantitativo. Participaram 216 crianças, entre 4 a 6 anos e 11 meses. Realizou-se entrevista com os pais/responsáveis e as crianças passaram por triagem visual, avaliação auditiva (audiometria tonal método triagem, emissões otoacústicas e imitanciometria), aferição de peso e altura, e posturografia computadorizada Horus®. Utilizaram-se os softwares Anthro e Anthro Plus para classificação das curvas de crescimento por idade, sexo, altura, peso e índice de massa corporal. Analisaram-se as respostas pelos testes estatísticos não paramétrico Kruskal-Wallis, post hoc Dunn-Bonferroni para comparações par-a-par entre idades, sendo p<0,05.

**Resultados:**

A medida de peso pelo comprimento demonstrou relação com diferentes condições de exame aos 4 anos, principalmente para o dado de velocidade média. O mesmo dado, em diferentes condições de exame, mostrou relação com o índice de massa corporal aos 4 e 5 anos de idade entre as crianças classificadas com excesso de peso e àquelas abaixo do peso. As crianças consideradas abaixo do peso apresentaram maior instabilidade no equilíbrio corporal. Altura e peso mostraram relação para diferentes condições de exame nas três faixas etárias.

**Conclusão:**

Verificou-se associação entre as respostas na posturografia e as medidas corporais individuais (peso, altura e índice de massa corporal), as quais exerceram influência sobre a manutenção do equilíbrio corporal. Desta forma, sugere-se que essas medidas sejam analisadas e consideradas na avaliação com posturografia computadorizada em crianças de 4 a 6 anos.

## INTRODUÇÃO

A posturografia é uma ferramenta de avaliação que permite a realização de uma análise global do equilíbrio, pois integra condições de acesso a informações visuais, somatossensoriais e vestibulares, visando mensurar as oscilações do corpo^(1)^, quantificando de forma objetiva e precisa os parâmetros relevantes nos domínios de tempo e frequência na comparação inter pacientes^(1)^. É composta por uma plataforma de força que verifica as oscilações realizadas pelo sujeito posicionado sobre ela em relação ao centro de gravidade e do centro de pressão (CP)^(2)^.

Em adultos, as respostas obtidas na posturografia possuem relação com as caraterísticas antropométricas do sujeito, como medidas de altura e peso, possibilitando interferência nas respostas^(3)^. Isso demonstra que as diferenças na composição corporal do sujeito e outras variáveis podem influenciar nos resultados do exame^(4)^. Para investigações clínicas, é necessária a normatização e padronização dos procedimentos de avaliação, bem como deve-se ter cuidado na quantificação do equilíbrio corporal ao utilizar as medidas antropométricas^(3)^.

Na população infantil, existe uma correlação entre a idade e as medidas de altura e peso, do mesmo modo que, tem-se uma correlação positiva entre a idade e a magnitude de oscilação corporal^(5)^. Na infância, as medidas antropométricas podem interferir negativamente no controle do equilíbrio corporal, apesar de sua correlação não estar definida de forma explícita^(6)^.

Na avaliação com a posturografia computadorizada do tipo dinâmica, ocorrem associações entre o excesso de peso corporal e valores abaixo do esperado em diferentes condições do exame, indicando interferência das medidas antropométricas na resposta do equilíbrio postural em crianças de 6 a 7 anos^(7)^. A avaliação de crianças com sobrepeso e obesidade, entre 6 e 9 anos, por meio de posturografia dinâmica e plataforma de força, demonstrou déficit no equilíbrio postural, no qual os pesquisadores acreditam ter sido potencializado pelas variáveis de sexo, idade, nível de atividade física e características antropométricas^(8)^.

Sugere-se que em estudos de normatização da posturografia computadorizada sejam realizadas análises dos achados pelas medidas antropométricas^(9)^. As medidas corporais, como a altura e o peso, podem influenciar, além do fator idade, no desenvolvimento do equilíbrio na população infantil, pois ocorrem mudanças estruturais no corpo continuamente pelo crescimento, fazendo com que as entradas sensoriais para o sistema vestibular sejam atualizadas/modificadas ao longo da infância^(10)^.

Verificou-se na literatura científica que existe influência das medidas de peso corporal e altura sobre o equilíbrio corporal em crianças, entretanto, não foram encontrados estudos analisando a possibilidade de associação entre as medidas corporais em crianças e os achados na posturografia computadorizada Horus®, o que fundamenta a relevância do presente estudo. Assim, tem-se como objetivo verificar a existência de associação entre o equilíbrio corporal (por meio dos achados da posturografia computadorizada Horus®) e medidas corporais pelas curvas de crescimento por idade, sexo, altura, peso e índice de massa corporal (IMC) em crianças hígidas, de 4 a 6 anos e 11 meses de idade.

## MÉTODO

O estudo descrito é de natureza transversal, utilizando uma abordagem quantitativa. Foi aprovado pelo Comitê de Ética da Universidade Federal do Rio Grande do Sul, com o número de parecer 39835. Além disso, o estudo seguiu as normas e diretrizes éticas estabelecidas pela Resolução 466/12 do Conselho Nacional de Saúde, que regulamenta a pesquisa envolvendo seres humanos.

Durante o processo de recrutamento e participação no estudo, foram adotadas medidas rigorosas para garantir o consentimento informado dos participantes. Foi fornecido aos responsáveis pelas crianças o Termo de Consentimento Livre e Esclarecido, explicando detalhadamente os objetivos, procedimentos, riscos e benefícios da pesquisa. Esse termo foi assinado pelos responsáveis visando autorizar a participação da criança no estudo.

Adicionalmente, foi solicitado o aceite pelo Termo de Assentimento da Criança, permitindo àquelas crianças que já eram capazes de compreender o processo pudessem consentir individualmente, pela assinatura, quando aplicável. Além disso, foi obtido o consentimento para uso de imagem, assegurando que qualquer registro visual das crianças durante o estudo fosse utilizado conforme as diretrizes estabelecidas.

Em relação aos critérios de inclusão, as crianças deveriam ter entre 4 e 6 anos e 11 meses de idade, apresentar desenvolvimento típico conforme as etapas do desenvolvimento infantil e não apresentarem queixas otoneurológicas e/ou auditivas. As crianças que não puderam participar, ou seja, aquelas que se enquadravam nos critérios de exclusão, foram as que tinham peso corporal inferior a 15 kg; diagnóstico de síndromes, perda auditiva ou alterações otológicas; presença de anormalidades craniofaciais; queixas de cefaleias, vertigem ou cinetose; histórico de quedas frequentes ou presença de tontura; dificuldade visual e/ou comprometimento motor que pudesse interferir na realização da avaliação; alterações neurológicas, cognitivas e/ou transtornos psíquicos evidentes; uso de medicamentos com ação sobre o sistema vestibular ou sistema nervoso central; dificuldades em compreender ou, por qualquer razão, executar os procedimentos e concluir todas as etapas de avaliação.

O contato inicial seguiu pela realização da anamnese com os pais/responsáveis pela criança, em formato de autorrelato, visando o conhecimento do desenvolvimento infantil até o momento, bem como, a presença de diagnósticos e/ou transtornos prévios, verificação de possíveis queixas auditivas e/ou relacionadas ao equilíbrio corporal que pudessem ser motivo de exclusão para a participação na pesquisa. E referente ao desenvolvimento infantil, foram explorados detalhadamente os domínios de linguagem e fala, cognitivo, social, emocional, motor grosseiro e fino.

As medidas antropométricas de altura e peso foram aferidas por meio de fita métrica fixa na parede da sala de exames e por balança digital disponível no local, respectivamente, sendo o peso corporal confirmado posteriormente pela balança de medição presente na plataforma Horus®. Visando a análise dessas medidas, os dados foram adicionados individualmente, para cada participante, no *software* Anthro^(11)^ para crianças com idade inferior a 5 anos e no *software* AnthroPlus^(12)^ naquelas com idade superior a 5 anos, ambos os programas disponibilizados gratuitamente, de forma *online*, pela Organização Mundial de Saúde (OMS). Para o cadastro e a análise dos dados, foi necessária também a adição manual dos dados de data de nascimento e data da medição.

Após a inclusão desses dados, os programas geram gráficos contendo as curvas de respostas correspondentes a cada umas das variáveis, como peso pela altura, peso pela idade, altura pela idade e IMC pela idade, no *software* Anthro^(11)^ e, para as variáveis peso pela idade, altura pela idade e IMC pela idade, no *software* AnthroPlus^(12)^. Desta forma, as respostas apresentadas em Desvio Padrão (DP) podem ser interpretadas por meio das diferentes cores presentes nas linhas dos gráficos: verde para normalidade (valor dentro da média da população), valor adequado; amarelo para DP -1 e DP +1 sendo considerado um sinal de alerta; vermelho para DP -2 e DP +2 quando refere-se a possível problema nutricional; preto para DP -3 e DP +3 pedindo a confirmação dos dados^(13)^ e roxo para implausibilidade biológica (IB). A IB ocorre quando a criança se encontra muito acima na altura e/ou peso para a idade, sugerindo-se como dado implausível para o sujeito cadastrado, entretanto, quando presente, as medidas da criança foram reavaliadas para confirmação do dado e, caso estivesse correto, manteve-se a classificação.

Para o índice de massa corporal (IMC) em crianças com até 5 anos, os dados são interpretados da seguinte forma: +1DP são descritas como risco de sobrepeso, acima de +2DP como sobrepeso, e acima de +3DP como obesas^(14)^; Para as crianças com idade acima de 5 anos, a interpretação é a seguinte: DP +1 refere-se a sobrepeso, DP +2 refere-se à obesidade, DP + 3 à obesidade grave, DP -1 para crianças abaixo do peso, DP -2 refere-se à magreza, DP -3 à magreza extrema e DP para valor dentro da normalidade^(13)^.

Segundo as orientações do manual de utilização do *Software* AnthroPlus^(13)^, foi necessário criar dois programas devido o valor do ponto de corte para a classificação de sobrepeso e obesidade, após análises das populações, serem diferentes nas faixas etárias nos dois grupos etários, idade inferior a 5 anos e faixa de 5 a 19 anos. A classificação do estado nutricional é realizada pela análise dos pontos de corte estabelecidos pela OMS para os índices de peso para idade, estatura para idade e IMC para idade^(14)^.

Na sequência, a bateria de testes que integraram as avaliações teve início pela avaliação do sistema auditivo periférico com a inspeção visual do meato acústico externo por meio do otoscópio da marca *Welch Allyn;* seguida de audiometria tonal método triagem, a qual consiste em varredura^(15)^ na intensidade de 20 dB nas frequências de 500, 1000, 2000 e 4000 Hz em ambas as orelhas, avaliadas individualmente. A audiometria foi realizada com a utilização do equipamento *Callisto* e do fone modelo TDH-39, estando a criança sentada confortavelmente em cadeira posicionada dentro de cabine acústica; Emissões otoacústicas transientes (EOAT) do tipo triagem, por meio do equipamento *AccuScreen* e utilização de fones do tipo inserção; Timpanometria com sonda de 226Hz e pesquisa dos reflexos acústicos contralaterais nas frequências de 500, 1000, 2000 e 4000 Hz. Estes foram realizados com o equipamento *Zodiac e* utilização de fones do tipo inserção e sonda. Consideraram-se aptas aquelas crianças que apresentaram respostas presentes nas EOAT e em todas as frequências testadas na audiometria tonal, curva timpanométrica do tipo A e reflexos acústicos presentes em ambas as orelhas. Todos os equipamentos utilizados neste estudo estavam calibrados conforme as normas ISO 8253-1.

A avaliação do sistema visual foi realizada por meio de triagem da acuidade visual pelo optotipo direcional “E” de Snellen, seguindo as orientações fornecidas pelo Ministério da Saúde^(16)^. Este teste é realizado avaliando individualmente cada olho. Para isto, a criança fez uso de um tapa olho com ilustração infantil e de forma lúdica utilizou-se o termo: "como um pirata”. Solicitou-se que cobrisse primeiramente o olho esquerdo que não estava sendo avaliado no momento, e após cobrisse o olho direito, sendo observado e verificado pela examinadora se estava realmente coberto e sem pressão sobre os olhos. Para a avaliação, requisitou-se que a criança falasse ou apontasse em qual direção estava enxergando as "pernas da letra E", previamente explicado e demonstrado. A avaliação teve início mostrando-se as figuras de cima para baixo na folha, posicionada na parede da sala em distância conforme as orientações do Ministério da Saúde^(16)^, ou seja, foram apresentadas primeiramente as letras maiores para as menores. Considerou-se resposta dentro da faixa de visão, ou seja, as respostas corretas correspondentes à linha 10.

Posteriormente foi analisado o equilíbrio corporal, por meio da posturografia computadorizada Horus**^®^** , da empresa Contronic. Houve necessidade de adaptação da plataforma e do *software* pelo fabricante, especialmente para o presente estudo, devido à necessidade de adequação do peso mínimo aceitável para realização dos testes na faixa etária. Para execução da avaliação, a Plataforma de Força ficou conectada por USB a um *notebook* da marca *Asus*, modelo X450CA, o qual continha o *software* Horus**^®^** , exibindo e registrando todos os dados fornecidos. As imagens necessárias para a realização da avaliação foram apresentadas em uma televisão de 40 polegadas da marca *Sony,* em uma distância de 1 metro da plataforma e do ponto de posicionamento da criança, localizada de forma que a tela estivesse no nível da altura dos olhos. Quanto as medidas físicas de altura, a plataforma possui 5cm de altura e a almofada de espuma que possui 5cm de altura com D33 de densidade, a qual foi adicionada para a realização das condições de teste 3 à 7.

Em relação as orientações e posicionamento inicial: a criança ficou descalça, subiu na plataforma, permaneceu em pé, com os pés afastados e permaneceu imóvel, de forma o mais parada possível. Cabe destacar que os pés deveriam ficar alinhados na linha horizontal sob a plataforma, afastados simetricamente da linha anteroposterior, com o dedo hálux apontando entre 0 e 15 graus. Outrossim, foi orientada a permanecer parada, olhando para a televisão onde apareceriam imagens, e, por fim, quando avisada, deveria fechar os olhos. A avaliação completa na posturografia consistiu em oito condições de exame, com duração de 30 segundos cada, sendo a criança orientada de forma específica, em cada condição. A descrição das condições de avaliação pode ser observada no Quadro 1.

**Quadro 1:** Condições de avaliação do exame de Posturografia computadorizada Horus®

CONDIÇÃO	SISTEMA AVALIADO	ORIENTAÇÃO PARA EXECUÇÃO	
Limite de Estabilidade (LE) - Plataforma fixa, sem almofada	Gera a área de estabilidade	Subir na plataforma e inclinar o corpo para frente, voltando ao centro, inclinar para trás, voltando para centro, inclinar o corpo para a direita, voltando ao centro, inclinar o corpo para a esquerda, voltando ao centro e após repetir. Devendo ser realizado sem pressa, sem fazer movimento com os quadris ou ombros e sem retirar os pés da plataforma, movimentando apenas os tornozelos. Etapa mais demorada, devido a necessidade de aprendizado do movimento e repetições.	
Condição sensorial 1 (C1) - Plataforma fixa, sem almofada	Somatossensorial, visual e vestibular	Permanecer em pé com os olhos abertos, olhando para um ponto amarelo fixo com tamanho de 10% contra um fundo preto, evitando se movimentar.	
Condição sensorial 2 (C2) - Plataforma fixa, sem almofada	Somatossensorial e vestibular	Permanecer em pé com os olhos fechados sem se movimentar.	
Condição sensorial 3 (C3) - Plataforma com almofada	Somatossensorial e visual	Idem orientação da condição sensorial 1	
Condição sensorial 4 (C4) - Plataforma com almofada	Vestibular	Idem orientação da condição sensorial 2	
Condição sensorial 5 (C5) - Plataforma com almofada e conflito visual	Preferência visual, conflito entre sistemas visual e vestibular. Somatossensorial impreciso.	Permanecer em pé, sem se movimentar, olhando televisão e assistindo a um vídeo contendo imagem de barras nas cores preto e branco em efeito optocinético horizontal para a direita, na velocidade de 10%.	
Condição sensorial 6 (C6) - Plataforma com almofada e conflito visual	Preferência visual, conflito entre sistemas visual e vestibular. Somatossensorial impreciso.	Permanecer em pé, sem se movimentar, olhando para a televisão passando um vídeo contendo imagem de barras nas cores preto e branco em efeito optocinético horizontal para a esquerda, na velocidade de 10%.	
Condição sensorial 7 (C7) - Plataforma com almofada e conflito visual	Preferência visual, conflito entre sistemas visual e vestibular. Somatossensorial impreciso.	Permanecer em pé, sem se movimentar, olhando na televisão vídeo de um túnel composto por barras finas movimentando-se para frente na velocidade de 4%, sem rotação.	

Para facilitar a avaliação com este público, foi necessário utilizar algumas estratégias lúdicas, conforme a necessidade individual da criança no momento. Essas estratégias incluíram: convidar o acompanhante da criança a realizar uma imitação da atividade, posicionando-se ao lado da criança; utilizar um boneco “Sr. Batata” parado como estátua no chão ao lado da plataforma; contar o tempo em voz alta, em ordem crescente ou decrescente; sugerir que, nas condições de teste contendo apresentação de imagens que poderia aparecer algum animal ao final do tempo, sendo necessário manter-se imóvel para não assustar o animal e fazê-lo ir embora (por exemplo: um ponto amarelo fixo na tela da televisão na condição de exame 1 poderia se transformar em um pintinho ao final dos 30 segundos e/ou uma zebra poderia aparecer ao final do tempo na avaliação com apresentação de estímulo optocinético). Essas estratégias ajudaram a manter a atenção e a cooperação das crianças durante a avaliação, principalmente das crianças menores.

Para cada criança, esteve sempre presente uma segunda pessoa posicionada próxima a ela no exame, visando proporcionar segurança e confiança. Além disso, o estudo contou com duas avaliadoras para melhorar a confiabilidade dos achados, verificando as respostas e o posicionamento/comportamento da criança durante a avaliação na posturografia.

Ao término, com todas as condições de exame da posturografia avaliadas, os valores das oscilações posturais foram registrados pelo estabilograma no sentido de movimentação para a esquerda e para direita (médio-lateral) e para frente e para trás (anteroposterior). Os dados também foram registrados pelo estatocinesigrama, que documenta o deslocamento do corpo no CP para o eixo médio-lateral e em relação ao deslocamento do CP no eixo anteroposterior, com uma elipse de confiança de 95%.

Neste trabalho, foram elencados os seguintes dados obtidos na posturografia Horus® para estudo: a) Limite de Estabilidade (LE) refere-se à distância em que a criança conseguiu se movimentar em relação ao centro de gravidade sem ter que alterar a base de suporte; b) Área do LE que indica a região em que o corpo movimentou-se sem queda/desequilíbrio em mm^2^; c) Elipse de confiança (EC) que quantifica o deslocamento ou a dificuldade da criança em manter-se sem oscilação em relação ao CP, em mm^2^; d) Velocidade que representa a velocidade média em relação ao CP. Uma velocidade média menor (em mm/s) é interpretada como indicativo de melhor equilíbrio corporal; e) Equilíbrio Funcional Residual (EFR) é a região ainda disponível para a oscilação corporal com segurança, calculada pela relação entre a área do LE e a área da EC, expressa em porcentagem. Quanto maior o valor do EFR, melhor o equilíbrio corporal.

Caso fosse identificada alguma alteração em qualquer uma das etapas de avaliação, independentemente do exame realizado, os pais/responsáveis eram orientados e a criança foi encaminhada para atendimento especializado complementar.

Os dados registrados foram analisados pelo *software Statistical Package for Social Sciences (SPSS),* versão 22.0 para *Windows*. Os dados categóricos foram apresentados em frequência relativa, enquanto os dados quantitativos foram descritos pela média e desvio padrão. Para comparar as diferentes faixas etárias, foram utilizados os testes estatísticos não paramétricos Kruskal-Wallis e o teste post hoc de Dunn-Bonferroni para comparações par-a-par. As diferenças foram consideradas estatisticamente significativas quando p < 0,05.

## RESULTADOS

Inicialmente, participaram da pesquisa 231 crianças, entretanto, considerando os critérios de elegibilidade, a casuística final foi de 216 crianças. Destas, 77 tinham entre 4 anos e 4 anos e 11 meses, 69 entre 5 anos e 5 anos e 11 meses, e 70 estavam na faixa etária de 6 anos a 6 anos e 11 meses.

Referente aos valores antropométricos de altura, registrados em centímetros, e peso, em quilogramas, para cada faixa etária estão dispostos na Figura 1.

**Figura 1:** Média de peso, em quilogramas, e altura, em centímetros, das crianças participantes da pesquisa (n=216)

Legenda: Kg = quilograma; cm = centímetros

Verificou-se achado estatisticamente significativo na relação entre a medida de altura aos 4 anos de idade (p=0,029). Na análise da medida peso pelo comprimento com as respostas na posturografia, não houve diferença estatística para o limite da estabilidade. Entretanto, observou-se significância estatística nas três primeiras condições do exame em crianças de 4 anos, conforme descrito na Tabela 1.

**Tabela 1:** Dados da medida de peso pelo comprimento e as respostas nas diferentes condições do exame de posturografia em crianças de 4 anos (n=77)

	N	Média± D.P.	Mínimo	Percentil 05	Percentil 25	Mediana	Percentil 75	Percentil 95	Máximo	p-valor^*^	Pairwise**
CONDIÇÃO 1 - PLATAFORMA FIXA E OLHOS ABERTOS											
VELOCIDADE MÉDIA LATERAL mm/s											
DP (A)	48	10,4± 4,7	4,0	4,5	6,5	8,6	14,5	19,3	21,7	0,028	D≠E 0,028
DP+1(B)	8	-4,1± 5,9	-11,0	-11,0	-10,1	-3,9	1,2	3,4	3,4		
DP+2(C)	6	8,4± 2,5	5,6	5,6	7,1	7,5	10,2	12,5	12,5		
IB (D)	11	7,1± 2,4	3,5	3,5	5,2	7,2	9,2	11,3	11,3		
DP-1 (E)	4	15,1± 4,1	10,5	10,5	12,0	14,9	18,3	20,2	20,2		
DESLOCAMENTO MÉDIO-LATERAL MÁXIMO PARA ESQUERDA mm											
DP (A)	48	-21,3± 13,3	-59,9	-48,5	-29,1	-21,6	-12,0	-2,7	2,2	0,024	C≠E 0,047
DP+1(B)	8	-19,5± 12,4	-37,7	-37,7	-30,1	-18,5	-9,4	-2,6	-2,6		
DP+2(C)	6	147,8±392,3	-26,1	-26,1	-18,2	-8,8	0,2	948,3	948,3		
IB (D)	11	-12,9± 10,4	-26,8	-26,8	-24,0	-12,6	-3,0	0,6	0,6		
DP-1 (E)	4	-33,7± 9,9	-47,9	-47,9	-39,7	-30,9	-27,7	-25,1	-25,1		
CONDIÇÃO 2 - PLATAFORMA FIXA E OLHOS FECHADOS											
ELIPSE DE CONFIANÇA mm^2^											
DP(A)	48	1016,9±634,1	100,5	244,7	574,3	894,9	1244,0	1957,5	3301,8	0,025	D≠E 0,041
DP+1(B)	8	995,9±845,4	223,0	223,0	493,7	725,2	1260,9	2784,4	2784,4		
DP+2(C)	6	693,9± 624,0	168,0	168,0	412,2	513,8	628,7	1926,7	1926,7		
IB (D)	11	598,0±385,3	224,1	224,1	299,7	449,3	854,7	1325,7	1325,7		
DP-1 (E)	4	1521,4±287,1	1181,7	1181,7	1283,7	1565,3	1759,1	1773,3	1773,3		
ELIPSE DE CONFIANÇA POR LIMITE DE ESTABILIDADE %											
DP(A)	48	12,5± 6,4	1,3	4,0	7,0	11,2	17,9	24,3	25,2	0,027	-
DP+1(B)	8	9,3± 3,9	5,2	5,2	6,5	8,5	12,0	15,7	15,7		
DP+2(C)	6	8,4± 5,7	2,3	2,3	2,8	8,3	11,3	17,6	17,6		
IB (D)	11	8,5± 5,4	2,9	2,9	3,5	7,7	9,6	21,6	21,6		
DP-1 (E)	4	19,2± 6,1	11,5	11,5	14,7	19,7	23,7	25,9	25,9		
VELOCIDADE MÉDIA LATERAL mm/s											
DP(A)	48	12,9± 4,9	3,5	5,8	8,8	12,4	16,8	19,9	28,3	0,002	C≠E 0,017D≠E 0,003
DP+1(B)	8	12,7± 5,5	6,8	6,8	10,0	11,5	13,2	25,2	25,2		
DP+2(C)	6	9,2± 2,8	5,7	5,7	6,7	9,0	11,4	13,3	13,3		
IB (D)	11	8,9± 3,0	5,6	5,6	6,6	8,1	9,9	15,8	15,8		
DP-1 (E)	4	22,5± 6,2	15,9	15,9	17,4	22,1	27,5	29,7	29,7		
VELOCIDADE MÉDIA ANTEROPOSTERIOR mm/s											
DP(A)	48	21,6± 6,4	9,8	11,5	16,4	22,1	25,3	32,5	35,5	0,028	D≠E 0,026
DP+1(B)	8	19,1± 4,1	14,4	14,4	16,0	18,4	22,0	25,7	25,7		
DP+2(C)	6	19,1± 6,1	12,5	12,5	14,2	18,1	22,6	29,4	29,4		
IB (D)	11	18,0± 5,1	11,2	11,2	15,2	17,1	21,0	29,7	29,7		
DP-1 (E)	4	29,7± 4,4	24,3	24,3	26,3	30,2	33,2	34,2	34,2		
EQUILÍBRIO FUNCIONAL RESIDUAL %											
DP(A)	48	87,5± 6,4	74,8	75,7	82,1	88,9	93,0	96,0	98,7	0,027	-
DP+1(B)	8	90,7± 3,9	84,3	84,3	88,1	91,6	93,5	94,8	94,8		
DP+2(C)	6	91,6± 5,7	82,4	82,4	88,7	91,8	97,2	97,7	97,7		
IB (D)	11	91,5± 5,4	78,4	78,4	90,4	92,3	96,5	97,1	97,1		
DP-1 (E)	4	80,8± 6,1	74,1	74,1	76,3	80,3	85,3	88,5	88,5		
VELOCIDADE MÉDIA TOTAL mm/s											
DP(A)	48	26,4± 8,5	10,2	12,9	20,7	25,3	31,8	41,8	45,3	0,011	D≠E 0,009
DP+1(B)	8	24,4± 7,2	15,9	15,9	20,2	23,0	26,9	39,3	39,3		
DP+2(C)	6	21,7± 7,0	14,4	14,4	15,2	20,8	25,9	33,1	33,1		
IB (D)	11	21,1± 6,3	13,1	13,1	17,6	19,2	23,1	35,9	35,9		
DP-1 (E)	4	39,8± 6,8	31,0	31,0	35,5	40,3	44,1	47,6	47,6		
CONDIÇÃO 3 - PLATAFORMA COM ALMOFADA E OLHOS ABERTOS											
VELOCIDADE MÉDIA LATERAL mm/s											
DP(A)	48	14,3± 4,8	5,6	7,0	11,1	13,5	18,3	22,7	25,4	0,017	C≠E 0,005
DP+1(B)	8	14,8± 5,6	8,5	8,5	10,2	13,2	20,1	23,0	23,0		
DP+2(C)	6	9,9± 3,3	5,3	5,3	6,5	11,0	11,2	14,1	14,1		
IB (D)	11	14,0± 3,9	7,8	7,8	10,5	13,0	17,7	20,4	20,4		
DP-1 (E)	4	21,4± 1,7	19,0	19,0	20,4	21,8	22,5	23,1	23,1		
VELOCIDADE MÉDIA TOTAL mm/s											
DP(A)	48	25,3± 7,6	9,5	12,5	20,6	23,3	30,0	39,7	42,4	0,032	C≠E 0,012
DP+1(B)	8	25,9± 9,0	14,6	14,6	19,1	23,9	33,4	39,6	39,6		
DP+2(C)	6	18,9± 5,5	12,3	12,3	13,3	19,1	23,6	26,0	26,0		
IB (D)	11	25,6± 4,9	19,9	19,9	22,2	23,8	30,3	34,4	34,4		
DP-1 (E)	4	36,5± 4,6	30,7	30,7	33,0	37,0	40,0	41,2	41,2		
ANÁLISE SENSORIAL PERCEPÇÃO VISUAL											
DP(A)	48	96,5± 6,1	68,6	88,5	93,3	96,4	100,9	104,2	105,8	0,04	C≠D 0,033
DP+1(B)	8	97,6± 3,5	94,2	94,2	94,8	96,0	101,0	102,7	102,7		
DP+2(C)	6	100,7± 4,9	94,4	94,4	97,5	99,9	105,3	107,2	107,2		
IB (D)	11	93,2± 3,8	85,5	85,5	90,8	94,6	96,1	98,0	98,0		
DP-1 (E)	4	97,4± 2,5	93,7	93,7	96,0	98,4	98,8	98,9	98,9		
ANÁLISE SENSORIAL PERCEPÇÃO VESTIBULAR											
DP(A)	48	89,7± 7,8	71,6	73,7	85,7	89,0	95,4	102,2	106,2	0,027	C≠D 0,029
DP+1(B)	8	90,8± 6,5	82,2	82,2	84,9	92,0	94,9	101,1	101,1		
DP+2(C)	6	93,9± 5,3	85,9	85,9	91,1	93,7	99,1	100,2	100,2		
IB (D)	11	83,5± 4,5	76,7	76,7	79,8	83,2	88,3	91,0	91,0		
DP-1 (E)	4	90,4± 11,0	78,5	78,5	81,2	90,8	99,7	101,5	101,5		

D.P. ao desvio padrão; p referente aos testes não paramétrico Kruskal-Wallis e o post hoc Dunn-Bonferroni para comparações par a par nas idades

* , ** referem-se a valores estatisticamente significativos

**Legenda:** N refere-se ao número de sujeitos; DP refere-se ao valor dentro do esperado para a idade; DP +1 refere-se a excesso de peso/ sobrepeso; DP +2 refere-se à obesidade; IB implausibilidade biológica, refere-se quando muito acima do esperado para idade; DP -1 refere-se a abaixo do peso esperado (conforme categorização pelo *software* Anthro^(11)^ da OMS)

Na análise da variável altura, houve diferença estatística para a C1 no CP médio-lateral (p=0,039, sendo DP +1 vs. normal p= 0,035), para C3 no CP anteroposterior (p=0,047, sendo DP +1 vs. DP +2 p= 0,036 e DP +2 vs. normal p= 0,032), para C4 na velocidade média médio-lateral (p=0,014, sendo DP +1 vs. normal p= 0,029), C5 no equilíbrio composto (p=0,008, sendo DP +1 vs. DP -1 p= 0,041, DP +2 vs. DP -1 p= 0,012, DP -1 vs. IB p= 0,03) e para velocidade média médio-lateral (p=0,011, sem relação par a par) e total (p= 0,022, sem relação par a par), C6 para velocidade média médio-lateral (p=0,01, sem relação par a par), anteroposterior (p=0,014, DP +1 vs. normal p= 0,013) e total (p= 0,006, DP +1 vs. normal p= 0,017), e C7 para as medidas de equilíbrio composto (p=0,007, sendo DP -1 vs. normal p= 0,037, DP -1 vs. DP +1 p= 0,009, DP -1 vs. IB p= 0,014), centro de pressão anteroposterior (p= 0,028, sendo DP +2 vs. normal p= 0,035, DP +1 vs. DP +2 p= 0,013), velocidade média médio-lateral (p=0,49, sem relação par a par).

A seguir, elencaram-se as respostas com resultados significativos por idade, conforme as variáveis de peso, altura e IMC:

Em relação ao peso**:** Aos 4 anos, foi observada diferença estatística para C4 na velocidade média anteroposterior (p=0,014, sem relação par a par) e para C7 no deslocamento médio-lateral máximo para a esquerda (p=0,05, sem relação par a par) e para a direita (p=0,023, sendo DP +2 vs. IB p= 0,033); aos 5 anos, houve diferença no LE (p=0,023, sendo DP -1 vs. normal p= 0,043 e DP -1 vs. IB p= 0,019) e para C5 na velocidade média médio-lateral (p=0,023, sem relação par a par).
Referente à altura: Aos 4 anos, houve resultado estatisticamente significativo para a análise sensorial vestibular (p=0,043 sem relação par a par), LE no deslocamento médio-lateral máximo para a direita (p= 0,006, sendo DP-1 vs. normal p= 0,018); aos 5 anos, os resultados foram significativos para a medida de LE (p=0,004, sem relação par a par), para C3 na velocidade média médio-lateral (p= 0,024, sendo DP +1 e DP -1 p=0,040) e no deslocamento máximo lateral para esquerda (p= 0,048, sem relação par a par), para C4 na velocidade média médio-lateral (p= 0,025, sem relação par a par), para C5 no equilíbrio composto (p= 0,028, sem relação par a par), e para C6 nas medidas de velocidade média médio-lateral (p= 0,035, sem relação par a par), anteroposterior (p= 0,025, sem relação par a par) e total (p= 0,015, sendo DP +1 vs. DP -1 p= 0,045); aos 6 anos, para a medida de LE (p=0,024, sem relação par a par), para C4 nas medidas de equilíbrio composto (p= 0,026 sem relação par a par) e velocidade média médio-lateral (p= 0,017, sendo DP +2 vs. normal p= 0,018) e total (p= 0,013, sendo DP +2 vs. normal p= 0,007), para C5 na velocidade média médio-lateral (p= 0,04 sem relação par a par), e para C6 na velocidade média médio-lateral (p= 0,019, sendo DP +1 vs. normal p= 0,024) e total (p= 0,031, sendo DP +1 vs. normal p= 0,022), e deslocamento médio-lateral máximo para direita (p= 0,011 sem relação par a par).
Em relação ao IMC: Não houve diferença significante em nenhuma das análises para crianças de 6 anos. No entanto, para as crianças de 5 anos, o IMC mostrou relação com a velocidade média total para o LE (p=0,05, sendo DP -1 vs. IB p=0,048). Para a faixa etária de 4 anos, foram observados mais resultados significativos, conforme pode-se observar na Tabela 2.

**Tabela 2:** Valores do índice de massa corporal e respostas nas diferentes condições de avaliação na posturografia em crianças de 4 anos (n=77)

	N	Média ± D.P.	Mínimo	Percentil 05	Percentil 25	Mediana	Percentil 75	Percentil 95	Máximo	p-valor*	Pairwise**
CONDIÇÃO 1 - PLATAFORMA FIXA E OLHOS ABERTOS											
VELOCIDADE MÉDIA LATERAL mm/s											
DP(A)	51	10,4± 4,6	4,0	4,2	6,3	9,2	14,4	19,3	21,7	0,033	D≠E 0,032
DP+1(B)	9	10,1± 4,1	4,7	4,7	7,6	8,4	12,4	16,5	16,5		
DP+2(C)	5	8,0± 2,6	5,6	5,6	7,1	7,2	7,4	12,5	12,5		
IB (D)	9	7,2± 2,3	3,5	3,5	6,2	7,2	7,5	11,3	11,3		
DP-1 (E)	3	16,7± 3,4	13,5	13,5	13,5	16,3	20,2	20,2	20,2		
DESLOCAMENTO MÉDIO MÁXIMO PARA ESQUERDA											
DP(A)	51	-21,7± 13,0	-59,9	-48,5	-29,4	-22,0	-12,6	-2,7	2,2	0,004	A≠C 0,023 C≠E 0,011
DP+1(B)	9	-18,5± 11,8	-37,7	-37,7	-25,9	-17,6	-10,9	-2,6	-2,6		
DP+2(C)	5	185,6± 426,4	-16,7	-16,7	-3,0	-0,8	0,2	948,3	948,3		
IB (D)	9	-13,1± 10,1	-24,0	-24,0	-21,7	-18,2	-3,5	0,6	0,6		
DP-1 (E)	3	-34,8± 11,8	-47,9	-47,9	-47,9	-31,5	-25,1	-25,1	-25,1		
CONDIÇÃO 2 - PLATAFORMA FIXA E OLHOS FECHADOS											
ELIPSE DE CONFIANÇA mm^2^											
DP(A)	51	1010,4± 622,9	100,5	244,7	567,0	885,8	1248,0	1957,5	3301,8	0,016	A≠D 0,046 D≠E 0,027
DP+1(B)	9	1013,8± 796,0	223,0	223,0	603,1	731,5	1212,5	2784,4	2784,4		
DP+2(C)	5	796,9± 637,1	401,7	401,7	480,2	547,4	628,7	1926,7	1926,7		
IB (D)	9	474,0± 359,7	168,0	168,0	275,9	312,5	498,5	1325,7	1325,7		
DP-1 (E)	3	1566,6± 333,7	1181,7	1181,7	1181,7	1744,9	1773,3	1773,3	1773,3		
ELIPSE DE CONFIANÇA POR LIMITE DE ESTABILIDADE %											
DP(A)	51	12,3± 6,1	1,3	4,0	7,0	10,6	17,6	24,0	25,2	0,026	D≠E 0,046
DP+1(B)	9	10,9± 6,2	5,2	5,2	6,6	8,2	14,8	24,3	24,3		
DP+2(C)	5	9,3± 5,6	2,8	2,8	6,9	7,7	11,3	17,6	17,6		
IB (D)	9	7,2± 5,9	2,3	2,3	3,4	6,8	8,5	21,6	21,6		
DP-1 (E)	3	19,6± 7,4	11,5	11,5	11,5	21,5	25,9	25,9	25,9		
CENTRO DE PRESSÃO ANTEROPOSTERIOR mm/s											
DP(A)	51	42,8± 12,8	8,8	23,2	36,2	41,0	48,5	64,3	82,2	0,048	A≠D 0,049
DP+1(B)	9	37,8± 5,6	30,1	30,1	34,7	37,4	39,2	50,0	50,0		
DP+2(C)	5	37,8± 4,7	32,3	32,3	35,4	36,7	40,1	44,7	44,7		
IB (D)	9	31,7± 12,2	17,4	17,4	21,4	32,5	34,9	57,2	57,2		
DP-1 (E)	3	46,6± 14,6	35,9	35,9	35,9	40,7	63,2	63,2	63,2		
VELOCIDADE MÉDIA LATERAL mm/s											
DP(A)	51	12,8± 4,9	3,5	5,8	8,7	12,1	17,0	19,9	28,3	0,003	A≠D 0,029 D≠E 0,004
DP+1(B)	9	13,2± 5,2	6,8	6,8	10,6	12,4	13,8	25,2	25,2		
DP+2(C)	5	9,9± 2,5	6,7	6,7	8,8	9,4	11,4	13,3	13,3		
IB (D)	9	8,2± 3,2	5,6	5,6	6,2	7,4	8,6	15,8	15,8		
DP-1 (E)	3	23,6± 7,0	15,9	15,9	15,9	25,3	29,7	29,7	29,7		
EQUILÍBRIO FUNCIONAL RESIDUAL %											
DP(A)	51	87,7± 6,1	74,8	76,0	82,4	89,4	93,0	96,0	98,7	0,026	D≠E 0,046
DP+1(B)	9	89,1± 6,2	75,7	75,7	85,2	91,8	93,4	94,8	94,8		
DP+2(C)	5	90,7± 5,6	82,4	82,4	88,7	92,3	93,1	97,2	97,2		
IB (D)	9	92,8± 5,9	78,4	78,4	91,5	93,2	96,6	97,7	97,7		
DP-1 (E)	3	80,4± 7,4	74,1	74,1	74,1	78,5	88,5	88,5	88,5		
VELOCIDADE MÉDIA TOTAL mm/s											
DP(A)	51	26,5± 8,5	10,2	12,9	21,1	25,2	31,7	41,8	45,3	0,02	D≠E 0,021
DP+1(B)	9	25,3± 7,2	15,9	15,9	22,3	23,7	29,3	39,3	39,3		
DP+2(C)	5	22,2± 7,5	14,4	14,4	17,0	20,5	25,9	33,1	33,1		
IB (D)	9	19,8± 6,5	13,1	13,1	17,6	18,5	20,1	35,9	35,9		
DP-1 (E)	3	39,5± 8,3	31,0	31,0	31,0	39,9	47,6	47,6	47,6		

DP ao desvio padrão; p referente aos testes não paramétrico Kruskal-Wallis e o post hoc Dunn-Bonferroni para comparações par a par nas idades

* , ** referem-se a valores estatisticamente significativos

**Legenda:** N refere-se ao número de sujeitos; DP refere-se ao valor dentro do esperado para a idade; DP +1 refere-se a excesso de peso/ sobrepeso; DP +2 refere-se à obesidade; IB implausibilidade biológica, refere-se quando muito acima do esperado para idade; DP -1 refere-se a abaixo do peso esperado (conforme categorização pelo *software* Anthro^(11)^ da OMS)

Quanto ao estado nutricional, analisado pelo *software* Anthro^(11)^ aos 4 anos, nas meninas, para a variável peso pelo comprimento, observou-se normalidade em 60,5% (n= 23), abaixo do peso em irmãs gêmeas 5,3% (n= 2), sobrepeso em 7,9% (n= 3), obesidade em 10,5% (n= 4) e incompatibilidade biológica em 15,8% (n= 6). Nos meninos, foi observada normalidade em 64,2% (n= 25), abaixo do peso em 5,1% (n= 2), sobrepeso em 12,8% (n= 5), obesidade em 5,1% (n= 2) e incompatibilidade biológica em 12,8% (n= 5).

No indicador IMC, foi observado normalidade em 68,4% (n= 26), abaixo do peso em 2,6% (n= 1), sobrepeso em 5,3% (n= 2), obesidade em 7,9% (n= 3) e incompatibilidade biológica em 15,8% (n=6) no sexo feminino. No sexo masculino, foi observada normalidade em 64,2% (n= 25), abaixo do peso em 5,1% (n= 2), sobrepeso em 17,9% (n= 7), obesidade em 5,1% (n= 2) e incompatibilidade biológica em 7,7% (n= 3). Isso demonstra que aos 4 anos, mais de um terço das crianças (n= 26) apresentaram IMC fora do esperado para a faixa etária.

O IMC, na idade de 5 anos, analisado pelo *software* AnthroPlus^(12)^, demonstrou que no sexo feminino, 42,9% (n= 15) das crianças classificadas como eutróficas (adequadas, faixa dentro da normalidade), 8,6% (n= 3) encontravam-se abaixo do peso, 31,4% (n= 11) sobrepeso, 11.4% (n= 4) foram classificadas como obesidade e 5,7% (n= 2) como incompatibilidade biológica. No sexo masculino, 58,8% (n= 20) estróficos, 3% (n= 1) apresentaram magreza, 14,7% (n= 5) sobrepeso, 20,5% (n= 7) obesidade e 3% (n= 1) incompatibilidade biológica. Em síntese, 50,2% (n = 34) das crianças aos 5 anos apresentaram IMC fora do esperado para a idade.

Para a faixa etária de 6 anos, o índice IMC (analisado pelo *software* AnthroPlus^(12)^) os resultados apresentados no sexo feminino foram: 35,3% (n= 12) eutróficas, 11,8% (n= 4) apresentaram peso abaixo do esperado, 29,4% (n= 10) sobrepeso, 20,6% (n= 7) obesidade e 2,9% (n= 1) 2,9% (n= 1) incompatibilidade biológica. No sexo masculino, 47,2% (n= 17) classificados como eutróficos, 2,8% (n= 1) abaixo do peso, 25% (n= 9) sobrepeso, 13,9% (n= 5) obesos e 11,1% (n= 4) como incompatibilidade biológica. Demonstrando que aos 6 anos, mais da metade das crianças, 58,6% (n = 41), apresentaram IMC fora do esperado para a idade.

## DISCUSSÃO

As alterações no controle postural podem gerar atrasos no desenvolvimento motor e na aprendizagem, resultando em interferência nas potencialidades relacionadas à linguagem, fala, escrita e leitura^(17)^. Isso reforça a necessidade dos padrões de normalidade bem definidos em equipamentos de avaliação, os quais considerem as particularidades da idade e variáveis individuais como medidas antropométricas e sexo. Tendo também como intuito agregar na prática clínica ao se utilizar uma ferramenta segura para o acompanhamento do desenvolvimento infantil.

Cabe salientar que a maturação durante o desenvolvimento ocorre respeitando uma ordem, não sendo diferente para a maturação dos sistemas sensoriais envolvidos no equilíbrio corporal, a qual ocorre primeiramente no sistema visual, após no sistema proprioceptivo e seguido do sistema vestibular, chegando na maturação funcional aos 9 anos de idade^(18)^. Adicionalmente, os ajustes posturais realizados pelo corpo mostram-se essenciais na função de compensar as perturbações recebidas durante os movimentos que necessitam serem coordenados no espaço. Nesse sentido, o reflexo vestíbulo-espinal (RVE) desempenha relevante atuação, pois permite coordenar a movimentação da cabeça e do pescoço com o movimento do tronco e do corpo, visando manter a cabeça na posição ereta e em tendo participação na estabilização postural do corpo globalmente mesmo na ausência de locomoção ativa^(19)^. O que destaca a relevância de pesquisas na área de Otoneurologia infantil, as quais busquem maior elucidação do desenvolvimento maturacional do equilíbrio corporal e das possíveis associações com as medidas antropométricas, bem como, por meio da utilização e estudo de equipamentos atualizados voltados para avaliação, diagnóstico e intervenção neste público.

A medida peso pelo comprimento é empregada para monitorar e acompanhar o crescimento e desenvolvimento de crianças até 5 anos^(14)^. No presente estudo, essa medida revelou uma associação significativa nas crianças de 4 anos com a velocidade média, bem como, respostas relacionadas aos sistemas visual e vestibular, possivelmente devido às diferenças estatísticas observadas na altura das crianças nessa faixa etária. Os valores de oscilação observados na velocidade média foram superiores nas crianças classificadas como DP-1, abaixo do peso, em comparação com aquelas classificadas como DP+2 e/ou IB, acima do peso. Supõe-se que seja devido à menor rapidez na movimentação corporal, o que pode estar relacionado ao aumento da massa corpórea e, consequentemente, à redução da oscilação de ajuste nas crianças consideradas mais pesadas. O que contribui para maior estabilidade do corpo em relação ao centro de pressão.

Estudo^(20)^ investigou a possível associação entre as respostas do centro de pressão em plataforma de força e a variável sobrepeso *versus* peso dentro do normal em crianças e adolescentes, na faixa etária de 7 a 12 anos. Os resultados revelaram que não houve diferença significativa entre os pares de mesma idade nas quatro condições sensoriais avaliadas. No entanto, houve maior variabilidade de respostas naquelas que encontravam-se acima do peso, para o dado de velocidade, mais especificamente para a velocidade médio-lateral^(20)^. No presente estudo, na primeira condição de exame, foi observada uma diferença estatisticamente significativa na velocidade médio-lateral entre os extremos DP-1 (baixo peso para a altura) e IB (criança com valores antropométricos muito acima do esperado para a faixa etária), sugerindo que peso e altura influenciam no equilíbrio das crianças aos 4 anos de idade.

Outro estudo^(21)^ realizado com crianças de 4 a 5 anos, demonstrou que o peso corporal não teve uma significância estatística nas respostas para os testes de marcha Tandem e de apoio unipodal, que avaliam a capacidade da criança de manter o equilíbrio em uma base de apoio estreita. No entanto, a mesma variável apresentou associação forte com as respostas na posturografia dinâmica e estática em 93 jovens do sexo masculino na faixa etária de 10 a 21 anos^(22)^. Os pesquisadores sugeriram que as alterações no equilíbrio observadas foram muito sutis para serem detectadas pelo exame, ademais, relatam como outra hipótese a redução da musculatura na composição corporal em relação ao peso, e não necessariamente por alterações na propriocepção^(22)^. Diferentemente do presente estudo, no qual foram encontrados resultados significativos em algumas das condições de exame para todas as faixas etárias, na análise do peso e respostas no equilíbrio corporal, demonstrando a influência desta variável para o controle da estabilidade corporal. Possivelmente, esses resultados discrepantes podem ser atribuídos às diferenças nos métodos de avaliação em comparação com o primeiro estudo e às diferenças na faixa etária dos participantes no segundo estudo.

Os resultados da presente pesquisa corroboram em parte com outro estudo^(23)^, que descreve uma associação entre as respostas obtidas em diferente plataforma de força com a medida peso, sendo o excesso de peso significativo para dos dados de frequência anteroposterior e de velocidade médio-lateral aos 8 anos de idade. Pesquisadores^(24)^ sugerem que o peso é um fator preditivo das respostas obtidas na avaliação do equilíbrio em crianças de 3 a 4 anos, conforme observado no Teste de Alcance Funcional, que visa identificar alterações dinâmicas no controle postural, deixando de ser um preditor aos 5 anos. Reitera-se a existência de uma relação entre o peso corporal e os achados na posturografia. No entanto, os achados da presente pesquisa diferem de outros estudos com população infantil, pois as crianças com peso abaixo do esperado foram as que apresentaram pior desempenho, demonstrando menor estabilidade corporal em comparação com aquelas que estavam acima do peso. Uma das hipóteses levantadas é a faixa etária pesquisada.

As medidas corporais de peso e a altura relacionam-se negativamente com o controle postural^(25)^. Embora seja desafiador encontrar material na literatura científica que exponha de forma explícita o impacto das medidas de altura e peso na estabilidade postural, pesquisadores perceberam uma correlação com dependência estatisticamente significante entre as medidas e os parâmetros analisados, tanto na condição de olhos abertos quanto fechados, em escolares de 6 a 9 anos^(26)^. A estatura pode interferir negativamente no controle postural de crianças^(6)^. Após analisar as respostas pelo teste Clínico Modificado de Integração Sensorial e os resultados na posturografia *Balance Master* para o LE, foi identificada associação entre os achados e as variáveis de idade, sexo, altura e IMC em crianças de 4 a 12 anos^(27)^. Os achados atuais corroboram essas descobertas, uma vez que foram encontradas diferenças estatisticamente significativas em diferentes condições de exame para as variáveis analisadas em todas as faixas etárias.

Aos 6 anos, idade em que mais da metade das crianças apresentaram IMC alterado, não foi encontrada relação entre o IMC e as condições do exame de posturografia. Entretanto, aos 5 anos de idade foi observada relação em apenas uma condição (velocidade) e para diversas variáveis aos 4 anos, especialmente para o dado de velocidade média. A relação entre a medida de velocidade média, que permite determinar a rapidez dos deslocamentos do centro de pressão na posturografia, foi observada diferença tanto aos 5 quanto aos 4 anos, corroborando com resultados de estudo anterior^(6)^. Esses achados são consistentes com uma revisão bibliográfica recente, que concluiu que o aumento na oscilação da estabilidade corporal está associado a um alto valor de IMC, destacando o peso como um forte preditor de alterações na estabilidade corporal, conforme publicações em diversos periódicos científicos^(28)^.

No presente estudo, observou-se que à medida que a idade avança, houve aumento do percentual de crianças com IMC inadequado. Em relação aos achados nutricionais associados ao IMC, pesquisadores na área de nutrição identificaram uma tendência ao excesso de peso em crianças de 5 a 9 anos que residem em uma cidade do interior do Rio Grande do Sul, reforçando a observação de desordens nutricionais na população do estudo^(29)^. Em outra cidade mais populosa do mesmo estado, um estudo diferente verificou que conforme houve o aumento da idade, também aumentava o número de crianças classificadas como obesas^(30)^. Houve uma prevalência maior de crianças acima do peso entre aquelas com idade igual ou superior a 5 anos em comparação com as mais jovens^(30)^.

Acredita-se que a falta de atividade, o aumento do tempo em casa, as limitações de espaços para circulação, somadas às restrições de convivência causadas pela pandemia do Coronavírus iniciada em 2019, possam ter contribuído para os achados, pois a coleta foi realizada no período de novembro de 2020 a abril de 2021. Fato que pode ser considerado uma das limitações desta pesquisa. Sugerem-se novas pesquisas envolvendo crianças na mesma faixa etária, especialmente aquelas com patologias e/ou queixas vestibulares, incorporando novos achados e avanços tecnológicos para uma avaliação cada vez mais precisa e abrangente.

Por fim, reitera-se que o equilíbrio corporal é um dos requisitos essenciais para a aprendizagem motora, realização de atividades diárias, adequação da interação social e do pleno desenvolvimento. A integração do sistema vestibular, com outros sistemas sensoriais, é importante para o adequado desenvolvimento de comportamentos espaciais complexos e habilidades cognitivas pela criança^(31,32)^. As alterações no equilíbrio corporal e postural podem restringir a adequada interação da criança com o meio, sendo capaz de interferir na aquisição e no desenvolvimento da linguagem e da aprendizagem, causando um impacto deletério na cognição, nas habilidades psicossociais e educacionais.

## CONCLUSÃO

Conclui-se, diante do exposto, que foi possível verificar a associação das medidas corporais individuais de peso, altura e IMC com a manutenção do equilíbrio corporal em crianças hígidas de 4 a 6 anos e 11 meses. Recomenda-se que tais medidas sejam analisadas e consideradas nos exames de posturografia computadorizada na população infantil, devido à sua influência significativa nos resultados obtidos.
